# Determination of the Dissociation Constant for Polyvalent Receptors Using ELISA: A Case of M13 Phages Displaying Troponin T-Specific Peptides

**DOI:** 10.1021/acsomega.3c02551

**Published:** 2023-07-12

**Authors:** Sebastian J. Machera, Joanna Niedziółka-Jönsson, Martin Jönsson-Niedziółka, Katarzyna Szot-Karpińska

**Affiliations:** Institute of Physical Chemistry, Polish Academy of Sciences, Kasprzaka 44/52, Warsaw 01-244, Poland

## Abstract

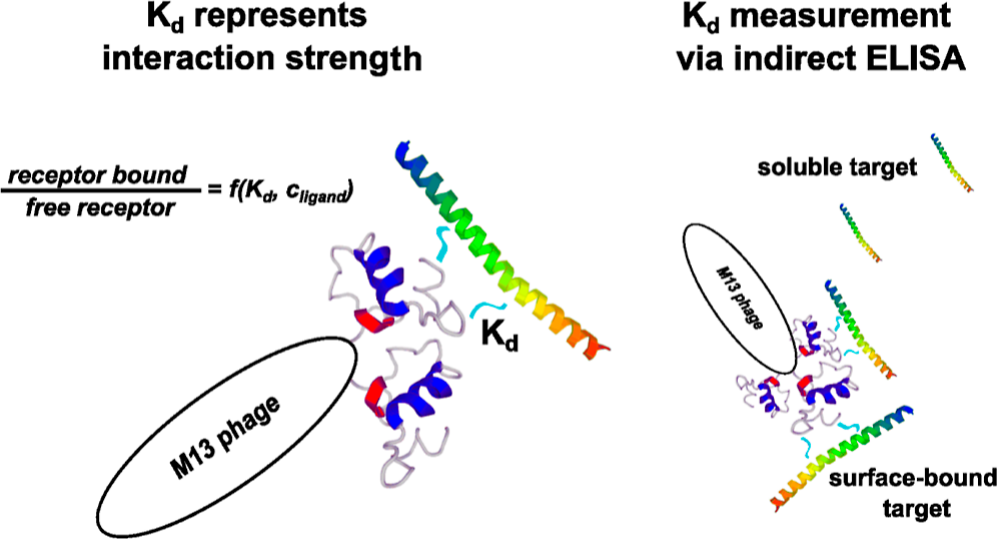

Phage-derived affinity peptides have become widespread thanks to their easy selection via phage display. Interactions between a target protein and its specific peptide are similar to those between antibodies and antigens. The strength of these non-covalent complexes may be described by the dissociation constant (*K*_d_). In this paper, protein-specific peptides are exposed on the pIII protein present in the M13 bacteriophage virion with up to five copies. Therefore, one phage particle can bind from one to five ligands. Here, we discuss the dependences between phage-displayed peptides and their ligands in solution using a model system based on troponin T (TnT) binding phages. Moreover, a method of calculating *K*_d_ values from ELISA experiments was developed and is presented. The determined *K*_d_ values are in the picomolar range.

## Introduction

Proteins are one of the most fundamental compounds that build living organisms. Various side chains of amino acids (mers in these biopolymers) enable multiple intra- and intermolecular interactions such as the Coulombic and van der Waals forces and aromatic π–π and ion-mediated salt bridges. Interactions between proteins are combinations of several attractive and repulsive forces between chemical groups of amino acids oriented properly to maximize attractive forces and minimize repulsive ones. Protein interactions often result in a conformational change in the tertiary protein structure, which regulates enzyme activity, opening channels or enabling motor activity. Understanding these interactions and their description measurably and comparably is a continuous aim of biochemistry and molecular biology.

The reaction dissociation constant is widely used to describe the strength of interactions between biomolecules. In the biochemical convention, as opposed to in physical chemistry, *K*_d_ is presented with a unit (usually mole per liter with an appropriate prefix). Typical values of *K*_d_ of immunological complexes are usually in the nanomolar or micromolar range. Generally, *K*_d_ is determined using surface plasmon resonance.^1,2^ But there are also other kinetic-based methods which are applied for *K*_d_ determination, like bio-layer interferometry,^1,3,4^ fluorescence correlation microscopy,^5^ thermal shift assay,^6^ nuclear magnetic resonance,^7^ gel chromatography,^8^ and electrochemical impedance spectroscopy.^9^

Enzyme-linked immunosorbent assay (ELISA) was initially proposed as an alternative to radioimmunosorbent test to examine antigen–antibody interactions.^10−12^ Later, this technique was developed and applied to the study of varied molecular interactions.^11^ In general, ELISA exhibits a low limit of detection, especially in its sandwich-like variant.^11^ There are a few variants of ELISA; the simplest are direct, indirect, sandwich, and reverse types. Nevertheless, thanks to a wide range of commercially available antibodies and kits, setting up an ELISA experiment is very flexible nowadays. In a typical ELISA, the signal (target–ligand binding) is amplified only by an enzymatic reaction. However, when M13 phages are used, the signal is amplified twice. Every five pIII-displayed ligands may interact with the target, and the binding of one phage particle via single ligand–target interaction provides about 2000 particles of pVIII protein (per virion) ready to be detected by secondary antibodies. In this way, phages provide about 10 000 fold (4 orders of magnitude) amplification of the signal, enabling detection of such a low phage amount as about 10^8^ plaque-forming units (pfu, the corresponding molar concentration of virions is about 10^–13^ M) administered into ELISA assay. ELISA may also be applied to study affinity between target and phage-displayed ligand (e.g., peptide).^13−18^

Indeed, the difference between monovalent binding affinity and multivalent binding avidity is well-known in the literature. However, in the case of phage–ligand complexes, the interactions between phage and ligands were described as 1:1 stoichiometry complexes,^16−18^ while they should be treated as polyvalent receptor-ligand systems, as presented in these studies. Antibodies are not monovalent because IgG and IgA immunoglobulins contain two active sites (antigen-binding sites), whereas IgM contains ten active sites exposed on the pentamer of dimers. Their ability to bind antigens is called avidity as opposed to affinity, which refers to the interactions between one antigen and one antigen-binding site. Omitting polyvalency of the receptor may lead to mistakes in the determination of the dissociation constant.^19^ The formation of non-covalent immunological (and immunological-like) complexes between peptides and proteins may be discussed as a reversible reaction, and a qualitative description could be presented based on chemical equilibrium laws.^20,21^ To the best of our knowledge, the mathematical model presented here is the first which enables the calculation of *K*_d_ (affinity) from ELISA under the assumption of phage polyvalency. However, the proposed model does not take into account the variability of the valency of M13 phage particles. The number of pIII protein copies per virion depends on several parameters. Empirically determined pIII number varies between studies according to the applied methodology.^22−25^

As *K*_d_ is widely used to express the strength of interaction between proteins, it may be used to describe the strength of target protein binding by phage-derived peptides obtained via phage display. This technique is based on the application of genetically modified bacteriophages (like the *Escherichia coli* M13 bacteriophage) exposing artificial amino acids sequences on their coat proteins.^26^ A mixture of various variants of exposed peptides (called a library) may be screened for variants with desired properties like binding to a target protein. Within our studies, we have obtained a specific peptide as a selective receptor for human cardiac troponin T (TnT). We tried to measure the strength of the interaction between phage-exposed peptide and TnT via ELISA. However, we found a gap in the literature devoted to studying the interactions between ligands and polyvalent receptors, especially discussing their mathematical and physicochemical basis. Troponin T (TnT), next to troponin C (TnC) and troponin I (TnI), is part of the troponin complex that plays an essential role in muscle contraction.^23^ Even though the troponin complex is present in skeletal and cardiac muscle (but not in smooth muscle), cardiac isoforms of TnT and TnI are tissue-specific and may be distinguished from their skeletal isoforms.^27^ These two troponins are released into the bloodstream from myocytes for several reasons, e.g., after long-lasting ischemia of the muscle region.^28^ Increased concentration of cardiac troponins is one of the fundaments of the fourth universal definition of myocardial infarction approved by several renowned cardiologist associations.^29^ Therefore, recognizing cardiac markers (TnT or TnI) in a highly efficient and selective manner is important for diagnosing myocardial infarction. Determination of *K*_d_ of isolated peptides enables a qualitative description of their affinity against TnT and, thus, the possibility of application in sensing devices.

This paper proposes a mathematical model of interactions occurring during ELISA between a ligand (troponin T) and a non-monovalent receptor (M13 bacteriophage particle exposing TnT-specific peptides). The provided equations enable the calculation of the dissociation constant from ELISA experiments with the critical impact of polyvalency of a phage, which was not discussed in any publications previously. The applied method may also cover other systems where monovalent molecules interact with multiple active sites of receptor-like natural antibodies. In addition, the interaction between phage-displayed peptides and TnT molecules was discussed according to their chemical structures.

## Results and Discussion

### Mathematical Model

The reaction between TnT and phage-displayed peptide may be treated as a reversible non-covalent complex formation.^20,21,30^ According to the induced fitting model, the interaction between the ligand and its binding site on the protein surface requires energy to deform structures and fit them in proper orientation and distance.^20^ However, if the interaction is strong enough, the energy released during the process of complex formation is higher than the energy required for complex dissociation. The reaction scheme of a reversible complex formation may be written as eq 1.
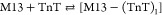
1

According to the mass action law, increasing ligand concentration causes a rise in receptor saturation. In the case of a monovalent receptor (complex stoichiometry 1:1), the reaction of receptor saturation shown in eq 1 is sufficient, while polyvalency of the receptor imposes the existence of multiple stages. In order to represent the saturation of *n*-valent receptor *n*, equations have to be written as eq 2, where *i* refers to subsequent numbers from 1 to *n*. The exact formulas for each stage are given in the Supporting Information (eqs S1–S5).
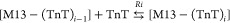
2

In an equilibrium state, formation and dissociation reactions occur at the same rate. Therefore, concentrations of particular reagents remain constant. The ratio of products and substrates concentrations (as approximations of activities) is constant under defined conditions (pH, ion strength, temperature) and is called a reaction constant. For the reaction depicted in eq 1, the reaction constant may be written as eq 3 where *K*_d_ refers to the dissociation constant.
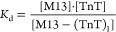
3

In the case of polyvalent receptor saturation, for each stage an equilibrium state is established and defined in eq 4.
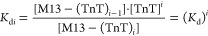
4

According to the geometry of phage particle pIII end, if all peptides are properly synthesized and exposed, they should not affect each other. Currently, there is no evidence that any allosteric effect might be present between peptides. Therefore, each stage dissociation constant equals *K*_d_ of the isolated TnT-peptide complex defined in eq 3. Moreover, the cumulative dissociation constant of particular stage saturation may be expressed as eq 4 where *K*_d_ refers to the dissociation constant of a single complex as eq 3.

For every case, the equation for TnT mass conservation may be written for both 1:1 and 1:*n* stoichiometry, as stated in eq 5.
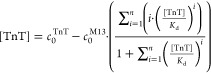
5

The free TnT equilibrium concentration ([TnT]) was expressed as a difference between total TnT concentration (*c*_0_^TnT^) and the sum of phage-bound TnT.

After the assumption of varying saturation levels and complex stoichiometry, each complex concentration may be expressed by total phage concentration (*c*_0_^M13^) and TnT-dependent saturation fractions. This equation is explained in the Supporting Information (eq S21 was obtained after combining S15, S17, and S19). In the case of the monovalent receptor, also presented by Friguet,^21^eq 5 may be written as eq 6.
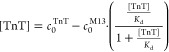
6

ELISA is a widely applied technique in biochemistry that enables the determination of biomolecule concentration.^11^ ELISA may be used to determine M13 phage concentration using TnT-coated wells and horseradish peroxidase (HRP)-conjugated anti-M13 antibodies.^16−18,21^ The signal is generated proportionally to the number of phages bound to surface-immobilized TnT. The presence of soluble TnT causes competition between this form and the surface-immobilized form, which leads to a signal decrease. If the signal measured with a predefined concentration of soluble TnT is called *a* and the signal of the same concentration of phages in the absence of TnT is *a*_0_, the fraction of occupied active sites (saturation) may be found (eq 7).
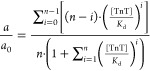
7

An explanation of the derivation of  ratios is presented in the Supporting Information (eqs S24–S27). Equation 7 is derived in the Supporting Information as eq S29. This approach (first proposed by Friguet^21^) enables the comparison of a signal from total phage active sites and partially saturated phages. The ratio  (eq 7) refers to the ratio of the signal recorded for partially occupied phage’s active sites in a mixture of TnT and phage (*a*) and the signal recorded in the absence of soluble TnT (*a*_0_) which may be interpreted as a signal from all active sites. Experimental determination of the  ratio is necessary to calculate the  ratio, which is crucial for further analysis.

Theoretically, a correlation between the concentration of phage-exposed active sites and a measured signal may be approximated in at least two ways. According to the first approach, the ELISA signal is linearly proportional to the concentration of phages exposing at least one active site or linearly proportional to the logarithm of the concentration of these forms (Supporting Information, eqs S28 and S30, respectively). The signal tends to be proportional to the logarithm of concentration in a wide range of concentrations and linear in a specified narrow range. On the other hand, the signal may be proportional to the total concentration of active sites, i.e., to the concentration of each TnT-phage complex ([M13-(TnT)_*i*_]) with an assumption of their stoichiometry (eq S29). Logarithmic interpretation of this case might also be considered (eq S31). Friguet’s model^21^ and the model presented in this work are focused on a linear approximation of the signal-concentration function. However, this work extends the original Friguet model with calculations for non-monovalent systems, which has not been analyzed earlier. To our knowledge, it is the first attempt to comprehensively study relations between a receptor (a phage exposing specific peptides) and ligands (target protein) at the equilibrium stage during an ELISA test. It is important to mention that only for linear approximation it is possible to present the  ratio independent from *c*_0_^M13^. This fact is a significant advantage due to difficulties in precise measuring of physical phage particle concentration. As a substitute, a biological titer of phages (in plaque-forming units per milliliter) is used in the literature and this work. Another attempt to develop a method enabling more accurate determination of phage concentration was spectrophotometrically measuring phages’ ssDNA in the sample.^31,32^ This method is not recommended for phage samples obtained via PEG precipitation.^33^ The true physical concentration may be determined via mass spectrometry^34^ under the assumption of a certain pVIII protein number per virion. Nevertheless, during ELISA, one also measures a signal from phage particles unable to infect bacteria and/or activate dye metabolism, which may cause a difference between physical phage concentration and biological titer. Also, Friguet’s original paper^21^ avoids the requirement of antibody concentration, which enables calculations for impure antibodies (with mixed specificity). The important assumption claiming that the signal measured in ELISA (*a*) is a linear function of the active sites (of antibodies in Friguet’s paper or phages in this work) was shown empirically in both works. Possible differences between linear and logarithmic interpretation are discussed in the Supporting Information (eqs S28–S31). Application of the discussed model in this case is possible only for linear approximation of concentration-signal dependence, as was also stated in Friguet’s work. The experiments presented in this work were designed to fulfil Friguet’s assumption, and therefore the obtained data does not allow examination of the logarithmic approach. A detailed discussion of the systems in which logarithmic approximation is preferred extends the frames of this investigation and may be studied in future work.

Calculation of the ratio  is possible analytically for 1:1 (eq 8) and 1:2 stoichiometry, but in the second case, expressions are very complicated (eq S35), so numerical methods are recommended to solve them. For higher valences, the ratios  have to be calculated numerically.
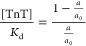
8

For determination of *K*_d_, calculated (analytically or numerically), the  value may be inserted into eq 5 or eq 6 after division by *K*_d_. Expressing  as a function of the total TnT concentration () enables calculation of *K*_d_ for monovalent (eq 9) and non-monovalent (eq 10) phages, respectively, as regression of the slope
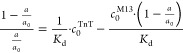
9
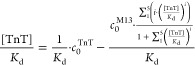
10

During an ELISA experiment, as proposed by Friguet,^21^ a constant concentration of phages is incubated with different concentrations of TnT. Saturation of phages (in solution) by soluble TnT limits the ability of phages to bind to immobilized TnT, and therefore, increasing concentration of TnT causes a decrease in signal from the phages, in our case, the M13. Theoretically, two options have to be discussed: (1) a signal (*a*) is generated for each phage with at least one unoccupied active site (eq 11) and (2) a signal (*a*) is generated proportionally to the total concentration of unoccupied active sites (eq 12).
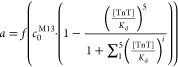
11
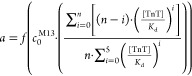
12

The first variant would be more probable for high TnT surface density (significantly higher capability than phages present in solution) where phages are mostly bound to the layer. According to the second variant, it is also possible that the recognition and binding process is slow enough to make the reaction kinetically limited. In this case, phages with a higher number of free active sites are more likely to be bound than more saturated ones. Nevertheless, the incubation times during ELISA and preincubation are comparable, and an equilibrium state can achieve equilibrium in both stages. More accurate assumptions should be proved empirically for each experimental system.

## Experimental Section

### Bio-Panning

In each bio-panning round, the input and output phases were titrated immediately before or after the experiment. The results of titrations are shown in Table 1. The presented mean *p*(O/I) value for each bio-panning round may be interpreted as the efficiency of phage binding. In chemical nomenclature, *p*(*x*) is defined as −log(*x*), so a higher *p*(O/I) means a lower ratio of phage output–input. The *p*(O/I) value for the first panning round is relatively low. However, usage of more concentrated Tween-20 (from 0.1 to 0.5%) causes a significant increase in *p*(O/I) values for the second round (around 4) despite a higher phage input. In addition, there is a difference between the second and the third *p*(O/I), which may be interpreted as an enrichment of TnT-binders in the phage pool, and it is the main aim of bio-panning via phage display. The increase in phage recovery yield is a widely reported observation by manufacturers and other authors.^14,17,35,36^

**Table 1:** Average Concentrations and Standard Deviations of Subsequent Phage Pools during Bio-Panning^a^

bio-panning round	mean input^b^	mean output^b^	mean O/I	mean *p*(O/I)
1st round	8.40 × 10^7^	(1.29 ± 0.68) × 10^5^	(1.54 ± 0.80) × 10^–3^	2.87 ± 0.21
2nd round	(6.15 ± 1.35) × 10^8^	(6.93 ± 3.29) × 10^4^	(1.10 ± 0.66) × 10^–4^	4.04 ± 0.28
3rd round	(2.30 ± 0.44) × 10^9^	(4.60 ± 1.34) × 10^5^	(2.22 ± 1.34) × 10^–4^	3.71 ± 0.21

a Output/input ratio (O/I) was calculated as a ratio of the number of phages used for panning and the number of recovered phages. *p*(O/I) = −log(O/I). The values of O/I and *p*(O/I) were previously calculated for each round, and mean values were calculated then.

b Phage titers are depicted in plaque-forming units calculated from the titer and volume of input (10 μL) or output phase (500 μL).

### Screening of Isolated Variants

**Figure 1:**
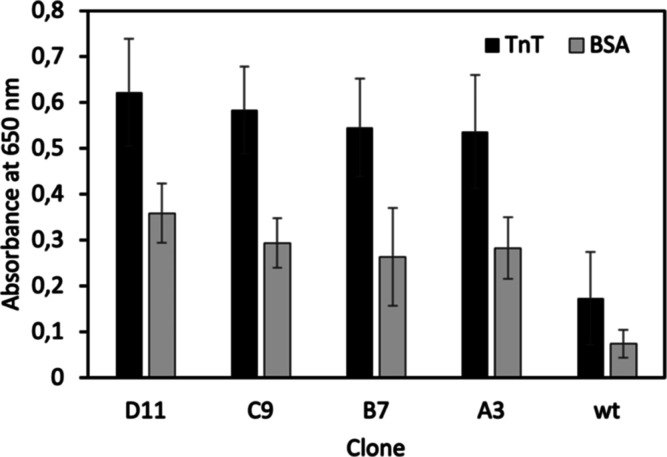
Screening of clones library against TnT and BSA (negative control). Error bars are calculated from the triple repeated experiments.

The best TnT binders were isolated via ELISA screening (Figure 1). As a negative control, wild-type M13 phage was used. Selected clones provide higher signals for TnT-containing wells than for bovine serum albumin (BSA, negative control). It is worth mentioning that both TnT- and BSA-precoated wells were blocked with the same 0.1% BSA solution, so BSA was present in each well in significant excess. Nevertheless, the presence of TnT causes a noticeable increase of signal, which may suggest a higher affinity of clones to TnT than BSA. Signals from wells that do not contain phages (blank sample) and wild-type phage, which does not expose any peptide, are at least 2 to 5 times lower than signals from the analyzed clones. This result confirms the impact of the displayed peptide on phage behavior against TnT and BSA. The significant drawback of this experiment is the inequality of concentrations of different clones because although all solutions were about 10^10^ pfu/mL, the exact value of the pre-exponential factor differed. Nevertheless, a comparison of the signal of each clone for TnT and BSA reveals a difference.

### Analysis of Exposed Peptides on the Selected TnT-Binding Phages

Sequencing of a fragment of gene encoding pIII minor coat protein showed the presence of 12-amino-acids peptide at the N-terminus in almost all phages. Simultaneous analysis of the sequenced variants (Table 2.) shows some conservative motifs in all four independent phage pools recovered after 3 rounds of bio-panning. The peptides DHAQRYGAGHSG (frequency 4 out of 16 proper sequences) and QMGFMTSPKHSV (frequency 8/16 proper sequences) are the most abundant. The isoelectric point (calculated using the online tool https://PepCalc.com/)^37^ of almost all sequenced peptides is above pH 7.4. The recognition steps were performed in PBS, making these peptides positively charged. This result may suggest that these peptides interact with TnT via electrostatic interactions because, in these conditions, the N-terminal region of TnT^27,38^ is negatively charged. In these peptides, predominantly hydrophilic amino acids with hydrogen bond donor groups are present, enabling them to interact with the glutamate-rich regions (which may act as hydrogen bond acceptors). Interaction with the negatively charged helical N-terminus may play a crucial role in recognizing the cardiac TnT isoform due to the tissue-specificity of this region.^27,38^ In conserved central and C-terminal TnT regions, there is a low number of aromatic amino acids (*Phe*: 6, *His*: 4, *Trp*: 3, *Tyr*: 4); however, this fact and the coincidence with the conservation of *His* residue in most of the peptides may suggest possible sites of interaction via π–π stacking. Additional studies are needed to prove the site where selected peptides may interact with TnT; however, the mentioned conserved positively charged peptides and *His* residue give hope that the site is located in the cardiac-specific N-terminal region.

**Table 2:** Amino Acid Sequences of Variants Selected for Analysis

clone name	frequency	amino acids sequence of selected clones												pI^a^
		1	2	3	4	5	6	7	8	9	10	11	12	
A3	4/16	D	H	A	Q	R	Y	G	A	G	H	S	G	7.93
B7	1/16	H	R	S	M	P	T	P	L	W	Y	T	A	9.59
C9	8/19	Q	M	G	F	M	T	S	P	K	H	S	V	9.84
D11	1/19	A	L	K	I	G	P	E	T	T	I	Y	M	5.99

a pI was calculated using the web tool https://PepCalc.com/.^37^

**Figure 2:**
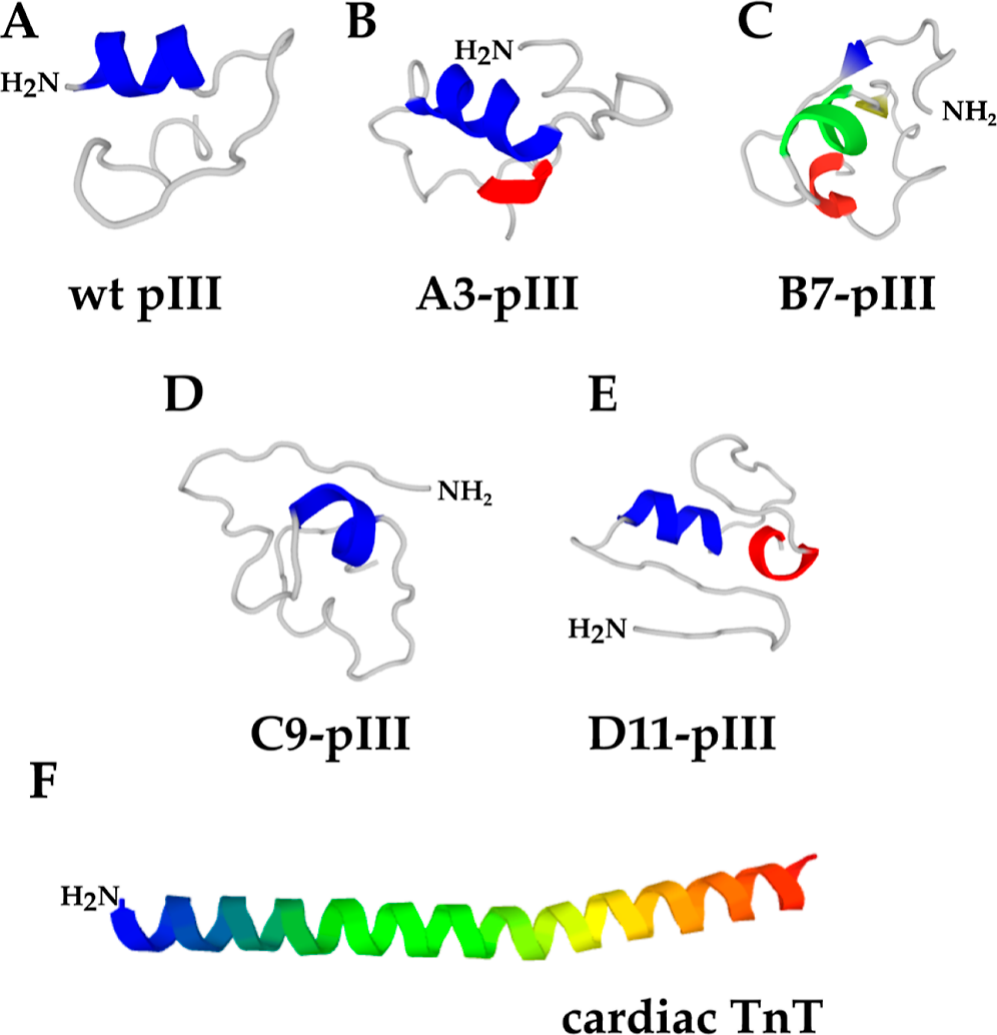
N-terminus of pIII protein: (A) wild-type pIII without additional peptide, (B) A3 clone (peptide sequence: DHAQRYGAGHSG), (C) B7 (QMGFMTSPKHSV), (D) C9 (ALKIGPETTIYM), (E) D11 (HRSMPTPLWYTA), and (F) N-terminal fragment of human cardiac isoform of TnT. Structures were generated using ref (39)–^41^.

In silico modeling of wild-type, pIII N-terminal 30-amino-acid fragment and several peptides fused at that site showed a probable secondary structure of the peptide and terminal region of pIII (Figure 2).^39−41^ The native helical fragments at N-terminus for the analyzed A3, C9, and D11 clones were quite affected. Nevertheless, undisturbed amplification and titration of these clones exclude the significant impact of additional fragments on bacteria recognition and infection, consistent with the literature reports.^42−44^ N-terminus of cardiac TnT is a long, helical structure exposing polar residues outside the helix, according to in silico simulation of appropriate sequence^45^ in PEP-FOLD3.^39−41^

### Phage Concentration Determination Using ELISA

Non-specific protein immobilization at the liquid/solid interface (of a microtiter plate) is one of the most commonly used methods of preparing wells for ELISA. This immobilized target is recognized by soluble molecules like antibodies or peptide-displaying phages. Stages of ELISA, which assumes specific recognition, may be described using a modified Langmuir-like isotherm.^46^ It should be noted that this model cannot be applied for initial non-specific precaution.^46^ Since wells are coated with TnT molecules and the number of available target molecules (active sites), they may bind a limited number of phages. Moreover, the number of bound phages limits the amount of bound HRP-conjugated antibodies. The surface density of HRP enzyme (i.e., the amount of HRP immobilized on the liquid/solid interface) is a crucial factor affecting the rate of substrate (3,3′,5,5′-tetramethylbenzidine, TMB) oxidation. The reaction rate may be expressed by the rate of the change of absorbance at 370 nm (which is the maximum absorption of the yellow byproduct^47^) under the assumption that the kinetics of the enzymatic reaction is zero order (Michaelis–Menten model and enzyme saturation). In this case, the measured signal is the enzyme activity defined as the slope of the linear time function of absorbance (eq 13). Enzyme activity is proportional to the amount of bound HRP-conjugated antibodies and indirectly to the number of bound phages (eq 14). When the reaction is stopped by the addition of sulfuric acid, the enzyme is denatured. As a result, the blue complex dissociates into a yellow product (adsorption maximum at 450 nm) and transparent TMB.^47,48^
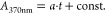
13
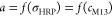
14

The application of kinetic-based ELISA (also shortened to k-ELISA) was previously described and developed by several authors.^49−52^ The signal recorded in this way has significant advantages such as independence from time-shifting, path length, wells material heterogeneity, and air bubbles.^53^ Single-data-point absorbance reading is easier and more high-throughput but generates a higher error.

**Figure 3:**
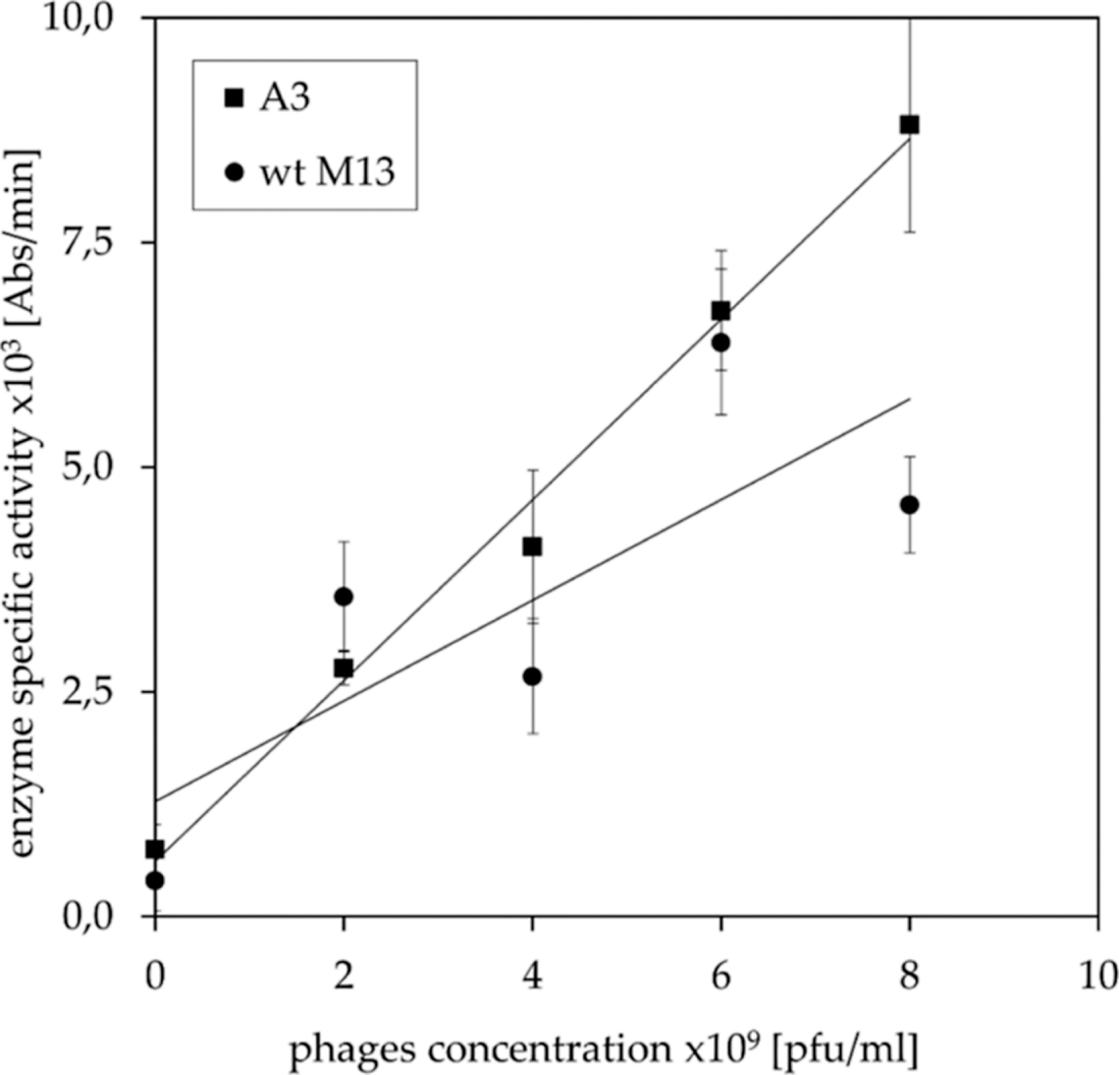
Calibration curve for detecting phages in sandwich k-ELISA using wells pre-coated with TnT. Experiments were performed in triplicate.

Figure 3 presents calibration curves for detecting the A3 variant (as an example) and wild-type M13 phage. Analysis for the A3 clone gave the calibration curve as *y* = 0.0010*x* + 0.0006 and *R*^2^ = 0.9961, indicating specific interaction with the studied target, while for the wild-type variant, it was *y* = 0.0006*x* + 0.0013 and *R*^2^ = 0.6323. Such a low *R*^2^ coefficient may suggest mainly non-specific interaction of the wild-type phage. For the A3, we also calculated sensitivity to be 0.264 × 10^9^ abs·mL·pfu^–1^·min^–1^. The empirically found range of the linearity of the signal enables the calculation of *K*_d_ via ELISA. In this range, d*a*/d(*c*_M13_) equals the constant value of sensitivity calculated above. The range of the linearity limits and indicates the range of analysis. To maximize the reproducibility of experiments, we decided to work with a phage concentration of about (1.0–10.0) × 10^9^ pfu/mL, which corresponds to about 1.6–16 pM.

### Determination of *K*_d_ via k-ELISA

ELISA (also k-ELISA) can be used to measure the dissociation constant (*K*_d_), which is commonly applied to describe affinity.^21^ According to the mathematical model presented above, the *K*_d_ value can be calculated via pre-incubation of a receptor with varied concentrations of ligand and subsequent detection of the soluble receptor (phage) via ELISA. This approach was adapted and applied to evaluate the affinity of several phage-derived ligands^16−18^ but without a theoretical analysis and fulfilling fundamental Friguet’s assumptions.^21^ In these papers, phages were treated as monovalent receptors while they are tri to pentavalent (due to a varied number of pIII protein per phage particle). Moreover, the authors applied a wide range of ligand concentrations, high excess compared to the receptor and treated the concentration-signal function as logarithmic, while the discussion above concluded that it is not possible to apply Frigeut’s method for almost completely saturated receptors (due to ligand excess).

In our work, analysis was performed for clones: A3, B7, D11, and wild-type M13. These clones were selected for further analysis because they exhibited the highest affinity in screening and could be easily amplified; therefore, the C9 clone was not chosen. The phages’ total concentration was about 1 × 10^10^ pfu/mL (16 pM) and was carefully determined using the biological method immediately before analysis. The range of ratios of total TnT and total phage concentration was 1:2, 1:1, 2:1, and 4:1. Increasing TnT concentration causes an increase in the saturation of phage-displayed receptors. As a result, a decrease in the ability to bind to immobilized TnT molecules occurs. Experimental data were analyzed according to the presented mathematical model. Calculated values of *K*_d_ (from linear slope regression) are listed in Table 3.

**Table 3:** Calculated Values of the Dissociation Constant for Different Assumed Stoichiometries^a^

clone	*K*_d_ [pM]		
	1:1	1:2	1:5
A3	115 ± 3	187 ± 2	172 ± 15
B7	77 ± 13	166 ± 30	143 ± 53
D11	167 ± 7	216 ± 40	115 ± 26

a All experiments were performed in triplicate.

For all phages, calculated *K*_d_ vary for different assumed complex stoichiometries. For the 1:1 stoichiometry, *K*_d_ values are the lowest (highest affinity). Then, they rise for bivalent receptors and slightly decrease for pentavalent. The M13 phages usually have three to five copies of the pIII protein.^25^ Therefore, the expected valency is between 2 to 5. For every variant, the difference between *K*_d_ calculated under the receptor’s mono- and bi-valency assumption is higher than the difference between the 1:2 and 1:5 stoichiometries. It suggests that the accurate stoichiometry is closer to 1:2 or 1:5 than to 1:1. The determined values are very low compared to typical *K*_d_ for immunological complexes. However, the presented method requires working under similar concentrations of M13 and TnT. The linear range of concentrations obtained for phages using k-ELISA was in the picomolar range; therefore, TnT concentration also had to be picomolar. A significant disadvantage of this method is that phage titer biologically measured may differ from the real physical concentration of phage particles in solution measured during ELISA.^54,55^ In the biological method, phages are only detected if they can infect bacteria and induce dye (x-gal) metabolism to a blue product. In solution, phages may form aggregates that produce false low phage titers from biological assays. For A3, B7, and D11 clones, values of *K*_d_ are in a similar range which does not correlate with their distribution in the recovered pool (Table 2). This fact may be explained by the varied rate of phages’ amplification in bacteria culture caused by the different proportions of amino acids in peptides and the possible interaction of peptides with bacterial proteins. The same explanation is suggested by the manufacturer of the Ph.D.-12 kit as justification for advising against the amplification of phage-displayed peptide libraries.^35^ For the wild-type M13, it was impossible to calculate a *K*_d_ that may have a physical sense (calculated *K*_d_ values were negative), which shows that the model may be applied only for specific interactions.

If a phage is treated as monovalent, the measured *K*_d_ does not refer to the thermodynamical strength of single peptide-TnT interactions. However, the resultant affinity (strength of single interaction) and valency (number of binding sites per particle) are known in immunology as avidity.^43,56,57^ Avidities are most significant for IgM antibodies that consist of five subunits presenting two antigen-binding domains on each one. As a result, a single IgM antibody can theoretically bind ten epitopes, causing 1:10 antibody–ligand complex stoichiometry. Moreover, bivalent IgG antibodies’ *K*_d_ is affected by non-monovalency in the same manner. Due to avidity’s different nature, compared to affinity, a relative dissociation constant maybe 10^2^ to 10^4^ lower than a single paratope-epitope *K*_d_. However, in several publications,^16−18^*K*_d_ was calculated without correction for non-monovalency of the antigen-binding particle (e.g., phage). One should be aware that the *K*_d_ calculated from ELISA in these papers is more avidity than affinity.

Several minor modifications of the presented method may be considered to improve the accuracy of *K*_d_ determination via ELISA. Limitation of non-specific interaction between phage particles and plate/surface-immobilized proteins and revision of phage concentration measurement would be the most significant improvements. However, the proposed mathematical interpretation of interactions between polyvalent receptors sheds new light on the field of affinity determination in phage-peptide systems, similar to the theory presented by the previous authors.^19^ The presented model was compared with empirical results that confirm the hypothesis about the mixed valency of phage particles depending on the number of pIII. Studies on peptide-protein complex formation and dissociation in other experimental systems may be discussed in a separate paper to provide comprehensiveness of the field of *K*_d_ determination in biochemical sciences.

The described method consumes low amounts of a target protein. Moreover, the phage material is easily amplified in bacteria culture. Owing to this, the presented assay is very suitable and valuable for research aimed at developing new high-affinity protein-specific receptors. The identified TnT-specific peptides might also find applications in research, especially connected with heart and cardiovascular diseases. Moreover, we anticipate that the selected TnT binding phages or peptides can be used to optimize a sensing layer toward implementing this concept for detecting the markers of cardiovascular diseases and transferring them into feasible applications in clinical diagnostics. The applied method may also cover other systems where monovalent molecules interact with multiple active sites of the receptor-like natural antibodies.

## Methods

Proteins (in the form of lyophilized powder): troponin T from the human heart, BSA and myoglobin from the equine heart (Mb) were purchased from Sigma (USA), dissolved in 100 mM phosphate-buffered saline pH = 7.4 (PBS) also bought from Sigma (USA) and stored at −20 °C. Ph.D.-12 Phage Display Peptide Library and *E. coli* ER2738 (F′ proA+ B+ lacIq Δ(lacZ) M15 zzf::Tn10(TetR)/fhuA2 glnV Δ(lac-proAB) thi-1 Δ(hsdS-mcrB)5. [rk– mk– McrBC−]) were bought from New England Biolabs (NEB, USA). Bacteria were grown in lysogeny broth (LB) bought from Roth (Germany), and plates were prepared by solidification of LB with 1,5% agar (Roth, Germany). Supplements: tetracycline (LabEmpire, Poland) to a final concentration of 5 μg/mL, IPTG (A&A Biotechnology, Poland) to 1 μg/mL, and x-gal (A&A Biotechnology, Poland) 0.8 μg/mL were added to sterile LB agar medium after autoclaving. PBST in an appropriate concentration was obtained by directly dissolving Tween-20 (Sigma-Aldrich, USA) in PBS. Dynabeads M-270 Epoxy was bought from Invitrogen/Thermo Fischer Scientific (USA). Isolation of phage DNA was performed as stated in ref,^58^ and sequencing of the phage genome was performed by Genomed (Poland) using −96pIII primer (NEB). Horseradish peroxidase-conjugated anti-M13pVIII monoclonal antibody was bought from Lab-Jot (NEB, USA) and used in 1:5000 dilution, while 3,3′,5,5′-tetramethylbenzidine (TMB, HRP enzyme substrate) was delivered by Thermo Fisher Scientific (USA) and used without pretreatment.

### General Bacteria and Phage Protocols and Bio-panning

Bacteria strains were stored in 50% glycerol solution at −80 °C. Bacteria were sown on LB plates containing 5 μg/mL of tetracycline and inoculated into fresh LB at least one day before experiments.

Phages were suspended in PBS solutions and stored at 4 °C. Titration was performed using the protocol described by Łoś et al.^59^ Briefly, immediately before usage 200 μL of bacteria culture was suspended in warm agar and split onto bottom agar containing x-gal and IPTG. Then, 2.5 μL of subsequent 10-fold dilutions of the phage sample were dripped onto the bacteria lawn. Prepared plates were incubated overnight at 37 °C. Phage titer was calculated by multiplying the number of single dots by the dilution factor and the constant coefficient for volume correction and depicted in plaque-forming units per milliliter (pfu/mL). Amplification and purification of phages were performed according to well-established protocols with slight modifications.^35^

Plate panning was performed regarding the general concept presented by Barbas et al.^60^ with some modifications. Polystyrene plates were precoated with TnT and incubated overnight (18–24 h) in 100 μL of 20 μg/mL TnT solution in PBS at 4 °C. Then, the wells were emptied and filled with 100 μL of 0.1% BSA solution (in PBS) and incubated for 2 h in the fridge. Later, they were washed four times with PBST 0.1% (in the first panning round) and 0.5% in the next two. For the first panning round, 100 μL of 100× diluted Ph.D.-12 Phage Display Peptide Library was used; however, it was tried immediately before use, showing an accurate phage concentration of 8.4 × 10^8^ pfu/mL. After 1 h of incubation on the shaker at room temperature, wells were washed four times using 0.1% (in the first round) or 0.5% (in further rounds) PBST. After washing, 100 μL of elution buffer (0.2 M glycine–HCl, pH 2.2) was added, and after 10 min it was collected. 10 μL was used to titrate the output phase, while 90 μL was directly transferred into the bacteria culture for amplification. Lysates (products of amplification and purification of phages) were titered and used for subsequent pannings or single clone isolation. This isolation was performed via whole-plate titration described by Łoś et al.,^59^ although the bottom agar contains x-gal/IPTG instead of antibiotics. Single clones (only blue dots) were picked up and placed in a standard amplification mixture.^35^

### Peptide Structure Prediction and Interaction Modeling

Three-dimensional structures of peptides exposed on the N-terminus of pIII protein were generated using PEP-FOLD3—a part of the online Mobyle system generously provided by Institute Pasteur and Paris Diderot University.^39−41^ Peptides were analyzed with 30 N-terminal amino acids of pIII after cleavage of the signal sequence (fragment: (X)_12_GGGAETVESCLAKPHTENSFTNVWKDDKTLDRY^35,61^) in order to maximize mimicking of the local environment of the peptide. Human cardiac TnT N-terminal fragment 2–50 (SDIEEVVEEYEEEEQEEAAVEEEEDWREDEDEQEEAAEEDAEAEAETEE^45^) without cleavaged *Met* was generated in the same manner.

### Enzyme-Linked Immunosorbent Assay

ELISAs were performed in 96-well polystyrene plates (Thermo Scientific, USA). Wells were precoated with the desired protein by 16–20 h incubation of the protein suspension in PBS at 4 °C. In all assays, 1:5000 dilution of HRP-antiM13pVIII monoclonal antibody conjugate was used. Signals were recorded using Synergy HTX Multi-Mode Reader by BioTek.

In the initial screening, plates were precoated with troponin T or BSA (negative control) by incubating 100 μL of 1 μg/mL appropriate protein solution. Then plates were blocked with 0.1% BSA for 2 h at 4 °C and washed 4 times with 0.1% PBST. Every isolated phage clone solution (phages concentration 10^10^ pfu/mL, 100 μL) was incubated with TnT and BSA for 1 h at 350 rpm at room temperature. Then wells were washed 4 times using 0.1% PBST, 100 μL of HRP-antibody conjugate was added, and plates were incubated as previously. After 1 h, plates were washed as before, and 50 μL of TMB was added. After 5 min, the reaction was stopped by adding 50 μL of 2 M sulfuric acid. Absorbance at 450 nm was read using a Synergy HTX Multi-Mode Reader (BioTek) plate reader.

The application of k-ELISA for measuring phage concentration was performed by precoating wells using 0.25 μg/mL TnT (or BSA in the negative control). The next steps regarding washing were performed in the same way as described above, despite 0.01% myoglobin being applied for blocking phages concentrations (range: 0, 2 × 10^9^, 4 × 10^9^, 6 × 10^9^, 8 × 10^9^ pfu/mL) and HRP-conj. Antibody solutions were added in amounts of 50 μL. Myoglobin was chosen as a blocking agent due to its small size (approximately 17 kDa), globular shape, and low price. BSA as a widely used blocking agent was avoided because it was present in all panning rounds, and peptides were suspected of exhibiting affinity to BSA. The signal was recorded by reading absorbance at 370 nm in a kinetic experiment every 70 s for 10 min.

The dissociation constant was calculated using k-ELISA, according to the procedure first described by Friguet.^21^ Phages (∼10^10^ pfu/mL corresponding to about 16 pM) were preincubated with different concentrations of TnT from 0 to 4 ng/mL (which corresponds to 108 pM). Next, mixtures were transferred into wells precoated with 0.25 μg/mL TnT (or BSA). Further steps were performed in the same manner as for phage concentration measuring experiments.

## Supporting Information Available

Extended version of the mathematical model of interactions between ligand (TnT) and its specific peptide-displaying M13 bacteriophages; theoretical aspects of the method of calculating dissociation constant from ELISA experiments; and all equations and assumptions described in details (PDF)
