# Supplementary material for: Determination of the Dissociation Constant for Polyvalent Receptors Using ELISA: A Case of M13 Phages Displaying Troponin T-Specific Peptides
Source: ACS Omega. 2023 Jul 12;8(29):26253–62. doi: 10.1021/acsomega.3c02551 (PMC10373194; doi:10.1021/acsomega.3c02551)
Supplement: Supplementary file 1 — ao3c02551_si_001.pdf [file ao3c02551_si_001.pdf]

SUPPLEMENTARY MATERIALS

Determination of the Dissociation Constant for Polyvalent Receptors using ELISA: a Case of M13 Phages Displaying Troponin T-specific Peptides

Sebastian J. Machera[a]*, Joanna Niedziółka-Jönsson[a], Martin Jönsson-Niedziółka[a] and Katarzyna Szot-Karpińska[a,]*

[a]Institute of Physical Chemistry, Polish Academy of Sciences, Kasprzaka 44/52, Warsaw, 01-244, Poland

* e-mail: sjmachera@gmail.com and kszot@ichf.edu.pl

Full mathematical model of calculation of K_d via indirect ELISA for non-monovalent TnT-specific phage particles

Part 1. Relations at equilibrium state

An immunological recognition occurs during antigen (TnT) incubation with the antibody-acting receptor (phage displaying TnT-specific peptides). During this process, proteins (or protein complexes like a phage capsid) interact via attractive and distractive forces and form an immunological complex if it is energetically favourable. This specific kind of 'chemical bond' has a non-covalent character, and its strength depends on fitting between dimensional structures.

The process of interactions of each phage-displayed peptide with TnT may be treated separately, assuming no steric hindrance between them. For simplification, it was assumed that each immunological complex was in order, not at the same time. As almost all non-covalent bond forming reaction is reversible, and after an appropriate time, the system achieves equilibrium. In this state, complex formation (association) and dissociation occur at the same rate, and concentrations of reagents remain constant.

In Eq.S1 it is presented the first stage of phage saturation. The depicted scheme refers to the interaction between completely unsaturated phage (M13) and soluble TnT molecules. It is the first stage of all discussed cases and the only reaction for monovalent receptor saturation. For non-monovalent receptors, one can write schemes of subsequent reactions. Several stages equals valency of the receptor, i.e. for n-valent phage; there are n stages represented by n reaction schemes shown in Eq.S1-S6. Eq.S6 represents expression for general cases. Reactions where $i > n$ in Eq.S6 do not have physical sense.

$$M13 + TnT \overset{R1}{\leftrightarrows} [M13 - (TnT)_1] \quad \text{(S1)}$$

$$[M13 - (TnT)_1] + TnT \overset{R2}{\leftrightarrows} [M13 - (TnT)_2] \quad \text{(S2)}$$

$$[M13 - (TnT)_2] + TnT \overset{R3}{\leftrightarrows} [M13 - (TnT)_3] \quad \text{(S3)}$$

$$[M13 - (TnT)_3] + TnT \overset{R4}{\leftrightarrows} [M13 - (TnT)_4] \quad \text{(S4)}$$

$$[M13 - (TnT)_4] + TnT \overset{R5}{\leftrightarrows} [M13 - (TnT)_5] \quad \text{(S5)}$$

$$[M13 - (TnT)_{i-1}] + TnT \overset{Ri}{\leftrightarrows} [M13 - (TnT)_i] \quad \text{(S6)}$$

If K_d refers to the dissociation constant of an isolated complex between TnT and phage-displayed peptide, then K_d may be treated as a cumulative dissociation constant for monovalent phage and equilibrium constant of the first stage of non-monovalent receptor saturation, as presented in Eq.S7. For the next stages of phage saturation (from 2 to n), it is possible to depict expressions of stage equilibrium constants that is shown in Eq.S8-S11 for reactions Eq.S2-S5, respectively. The general formula for i-step of saturation is written in Eq.S12.

$$K_d = K_{d1} = \frac{[M13] \cdot [TnT]}{[M13 - (TnT)_1]} \quad \text{(S7)}$$

$$K_{d2} = \frac{[M13 - (TnT)_1] \cdot [TnT]}{[M13 - (TnT)_2]} \quad \text{(S8)}$$

$$K_{d3} = \frac{[M13 - (TnT)_2] \cdot [TnT]}{[M13 - (TnT)_3]} \quad \text{(S9)}$$

$$K_{d4} = \frac{[M13-(TnT)_3]\cdot[TnT]}{[M13-(TnT)_4]} \quad (S10)$$

$$K_{d5} = \frac{[M13-(TnT)_4]\cdot[TnT]}{[M13-(TnT)_5]} \quad (S11)$$

$$K_{di} = \frac{[M13-(TnT)_{i-1}]\cdot[TnT]}{[M13-(TnT)_i]} \quad (S12)$$

Expression of the cumulative dissociation constant may be determined by transforming and substituting stage dissociation constants. At first, each stage's the more saturated complex concentration ($[M13-(TnT)_i]$) is defined as the function of this stage dissociation constant, $[TnT]$ and less saturated complex ($[M13-(TnT)_{i-1}]$) by simple transformation of the appropriate equation from Eq.S8 to Eq.S11 as it was presented in Eq.S13 for the general formula from Eq.S12. These transformations and substitutions were repeated i-times to represent K_{dn} as a function of subsequent K_{di}, $[TnT]$ and $[M13]$ what, according to the assumption from the previous paragraph, refers to K_d (Eq.S14). After the assumption that peptides exposed on the surface of phage pIII proteins do not interfere, stage dissociation constants may be treated as equal, and therefore cumulative dissociation constant of n stages equals K_d raised to the nth power (Eq.S15).

$$[M13-(TnT)_i] = [M13-(TnT)_{i-1}]\cdot\frac{[TnT]}{K_{di}} \quad (S13)$$

$$K_{dn} = \prod_{i=2}^{n} K_{di}\cdot\frac{[M13]\cdot[TnT]}{[M13-(TnT)_1]} = \prod_{i=1}^{n} K_{di} \quad (S14)$$

$$K_{dn} = \frac{[M13]\cdot[TnT]^n}{[M13-(TnT)_n]} = (K_d)^n \quad (S15)$$

At all stages of the reaction (before and after achieving equilibrium) the total concentration of phages and TnT remains constant, which is a direct consequence of the mass conservation law. The mass conservation equation for phages is shown in Eq.S16, whereas in Eq.S17, it is presented after applying Eq.S14.

$$c_0^{M13} = [M13] + \sum_{i=1}^{n}[[M13-(TnT)_i]] \quad (S16)$$

$$c_0^{M13} = [M13] + \sum_{i=1}^{n}\left([M13]\cdot\left(\frac{[TnT]}{K_d}\right)^i\right) = [M13]\cdot\left(1+\sum_{i=1}^{n}\left(\frac{[TnT]}{K_d}\right)^i\right) \quad (S17)$$

For the phage population, it is possible to define the fraction of a particular form of phages (i.e., free or bound with a specified number of ligands) by simple transformation of Eq.S16 and Eq.S17. This fraction, α_i, may be defined as unbound phages (α_{M13}), phages saturated with i TnT molecules ($\alpha_{[M13-(TnT)_i]}$) and completely saturated phages ($\alpha_{sat} = a_{i=n}$) are defined in Eq.S18, Eq.S19 and Eq.S20, respectively. Thank these operations, concentrations of particular phage forms are functions of total phages concentration (c_0^{M13}), free TnT concentrations ($[TnT]$) and K_d value, and they may be calculated in this manner.

$$\alpha_{M13} = \frac{[M13]}{c_0^{M13}} = \frac{1}{1 + \sum_{i=1}^{n} \left(\frac{[TnT]}{K_d} \right)^i} \quad \text{(S18)}$$

$$\alpha_{[M13-(TnT)_i]} = \frac{[M13 - (TnT)_i]}{c_0^{M13}} = c_0^{M13} \cdot \left(\frac{\left(\frac{[TnT]}{K_d} \right)^i}{1 + \sum_{i=1}^{n} \left(\frac{[TnT]}{K_d} \right)^i} \right) \quad \text{(S19)}$$

$$\alpha_{sat} = \frac{[M13 - (TnT)_n]}{c_0^{M13}} = \frac{\left(\frac{[TnT]}{K_d} \right)^n}{1 + \sum_{i=1}^{n} \left(\frac{[TnT]}{K_d} \right)^i} \quad \text{(S20)}$$

Similarly, the TnT mass balance may be written as in Eq.S21, where a change in $[TnT]$ is caused by binding to phage-displayed peptides, and appropriate stoichiometry must be assumed, i.e. in the complex $[M13 - (TnT)_i]$ there are bound i TnT molecules.

$$c_0^{TnT} = [TnT] + c_0^{M13} \cdot \left(\frac{\sum_{i=1}^{n} \left(i \cdot \left(\frac{[TnT]}{K_d} \right)^i \right)}{1 + \sum_{i=1}^{n} \left(\frac{[TnT]}{K_d} \right)^i} \right) \quad \text{(S21)}$$

According to presented formulas one can calculate the concentration of unoccupied pIII-exposed peptides (active sites) in the sample. The initial active site concentration (c_{pep}) is proportional to the number of peptides per virion (n).

$$c_{pep} = c_0^{M13} \cdot \sum_{i=0}^{n} \left((n - i) \cdot [M13 - (TnT)_i] \right) = c_0^{M13} \cdot \sum_{i=0}^{n} \left((n - i) \cdot \left(\frac{[TnT]}{K_d} \right)^i \right) \quad \text{(S22)}$$

In the general case, Eq. S21 after dividing by K_d and transformation may be presented as in Eq. S23. The below equation combines the most crucial parameters required for the description of equilibrium in non-monovalent complexes: K_d, $[TnT]$ and c_0^{TnT}.

$$\frac{[TnT]}{K_d} = \frac{c_0^{TnT}}{K_d} - \frac{c_0^{M13} \cdot \left(\frac{\sum_{i=1}^{n} \left(i \cdot \left(\frac{[TnT]}{K_d} \right)^i \right)}{1 + \sum_{i=1}^{n} \left(\frac{[TnT]}{K_d} \right)^i} \right)}{K_d} \quad \text{(S23)}$$

Part 2. Indirect ELISA

Equation S23 cannot be solved without knowing three of the four of these parameters: K_d, $[TnT]$, c_0^{TnT} or c_0^{M13}. For this purpose, an indirect enzyme-linked immunosorbent assay (ELISA) experiment may be

performed where the equilibrium concentration of M13 ($[M13]$) is directly measured. In the discussed ELISA system, phages are pre-incubated with soluble TnT until the achievement of equilibrium. In the next step, the equilibrium mixture is incubated in a TnT-coated polystyrene plate. Suspended phages recognize immobilized TnT (they act as primary antibodies), and then HPR-conjugated anti-M13pVIII mAb is added to produce a colourimetric signal after catalysis of TMB oxidation. Phages bind to immobilized TnT via pIII-displayed peptides. Due to pre-incubation with TnT, some phages are at least partially saturated, and their ability to bind to the immobilized target is decreased.

In regular ELISA, the signal is dependent on the concentration of the analyte. Usually, the signal is proportional to the logarithm of the analyte concentration ($s \propto \log(c)$) in a wide range. In a narrow range (discussed in detail in this work), the signal may be approximated by a linear signal ($s \propto \mathrm{c}$). In the discussed ELISA, two variants are possible: (1) the signal (a) is proportional to the concentration of phage particles that have at least one unoccupied pIII-exposed peptide (Eq. S24), or (2) a is proportional to the concentration of unoccupied active sites (peptides) as shown in Eq. S26. The reference signal (a_0) refers to the signal measured in absence of TnT (all active sites unoccupied) (Eq. S25 and S27).

$$a = f([M13] - [M13 - (TnT)_n]) = f\left(c_0^{M13} \cdot (1 - \alpha_{sat})\right) \tag{S24}$$

$$a_o = f\left(c_{pep}^0\right) = c_0^{M13} \tag{S25}$$

$$a = f\left(c_{pep}\right) = f\left(c_0^{M13} \cdot \left(\sum_{i=0}^{n} ((n-i) \cdot [M13 - (TnT)_i])\right)\right) \tag{S26}$$

$$a_o = f\left(c_{pep}^0\right) = n \cdot c_0^{M13} \tag{S27}$$

The first variant would be favourable if the first ELISA recognition step was an equilibrium process, and the monolayer capacity was higher than the phage amount during the assay. In contrast, the second option is kinetics-based and assumes that phage particles with a higher number of free active sites show a higher probability of recognizing immobilized TnT. The mathematical expression of both variants (ratio of the signal measured during assay with specified TnT concentration and signal without TnT) are shown in Eq. S28 and Eq. S29 for the first and second variants, respectively. In Eq. S30-S31 there are interpretations of Eq. S28-S29 for logarithmic concentration-signal determination. Equations S28-S31 were derived after assumption that when calculating $a = f(c), f(0) = 0$.

This study is focused on the linear concentration-signal dependence system and assumes that the signal measured in ELISA is proportional to the total number of active sites, which is presented in Eq. S26. As is mentioned in the main article, studying of the rest of possibilities requires further research and exploration of the model in that way. In Eq. S29 and S31, the upper range limit equals $n - 1$ because for $i = n$ the phage is completely saturated with ligands, and so would not be able to bind the target. Thus:

$$\frac{a}{a_0} = 1 - \frac{\left(\frac{[TnT]}{K_d}\right)^n}{1 + \sum_{i=1}^{n} \left(\frac{[TnT]}{K_d}\right)^i} \tag{S28}$$

$$\frac{a}{a_0} = \frac{\sum_{i=0}^{n-1} (n-i) \cdot \left(\frac{[TnT]}{K_d}\right)^i}{n \cdot \left(1 + \sum_{i=1}^{n} \left(\frac{[TnT]}{K_d}\right)^i\right)} \tag{S29}$$

$$\frac{a}{a_0} = 1 - \frac{\log\left[c_0^{M13}\left(\frac{\left(\frac{[TnT]}{K_d}\right)^i}{1 + \sum_{i=1}^{n}\left(\frac{[TnT]}{K_d}\right)^i}\right)\right]}{\log\left(c_0^{M13}\right)} \tag{S30}$$

$$\frac{a}{a_0} = \frac{\sum_{i=0}^{n-1}\left[\log\left[c_0^{M13} \cdot \left(\frac{(n-i) \cdot \left(\frac{[TnT]}{K_d}\right)^i}{n \cdot \left(1 + \sum_{i=1}^{n}\left(\frac{[TnT]}{K_d}\right)^i\right)}\right)\right]\right]}{\log\left(c_0^{M13}\right)} \tag{S31}$$

For each experiment $\frac{a}{a_0}$ may be found and $\frac{[TnT]}{K_d}$ may be calculated analytically (only for 1:1 system) or numerically. In the simplest case of a 1:1 stoichiometry, $\frac{a}{a_0}$ may be expressed as in Eq. S32, and after transformation, $\frac{[TnT]}{K_d}$ is easily presented in Eq. S33. In this system, both models represented in Eq. S28-S29 are equal.

$$\frac{a}{a_0} = \frac{1}{1 + \frac{[TnT]}{K_d}} \tag{S32}$$

$$\frac{[TnT]}{K_d} = \frac{1 - \frac{a}{a_0}}{\frac{a}{a_0}} \tag{S33}$$

Equation S23 for $n = 1$ may be expressed as follows in Eq. S34 where the TnT mass balance was extended with Eq. S32. In the same manner, Eq. S23 may be written for 1:2 stoichiometry; however, TnT mass balance has to be calculated from an appropriate interpretation of Eq. S21.

$$\frac{1 - \frac{a}{a_0}}{\frac{a}{a_0}} = \frac{c_0^{TnT}}{K_d} - \frac{c_0^{M13} \cdot \left(1 - \frac{a}{a_0}\right)}{K_d} \tag{S34}$$

$$\frac{1-2\cdot\left(\frac{a}{a_0}\right)+\sqrt{1-12\cdot\left(\frac{a}{a_0}\right)\cdot\left(\left(\frac{a}{a_0}\right)+1\right)}}{4\cdot\left(\frac{a}{a_0}\right)}=\frac{c_0^{TnT}}{K_d}-\frac{c_0^{M13}\cdot\left(\frac{2\cdot\left(\frac{a}{a_0}\right)\cdot\left(4\cdot\left(\frac{a}{a_0}\right)^2-8\cdot\left(\frac{a}{a_0}\right)+(a-1)\cdot\sqrt{1-12\cdot\left(\frac{a}{a_0}\right)\cdot\left(\left(\frac{a}{a_0}\right)+1\right)}+1\right)}{12\cdot\left(\frac{a}{a_0}\right)^2-8\cdot\left(\frac{a}{a_0}\right)+(a-1)\cdot\sqrt{1-12\cdot\left(\frac{a}{a_0}\right)\cdot\left(\left(\frac{a}{a_0}\right)+1\right)}+1}\right)}{K_d} \quad \text{(S35)}$$

In other cases, $\frac{[TnT]}{K_d}$ must be found numerically, and then the regression of the slope $\frac{[TnT]}{K_d}=f\left(c_0^{TnT}\right)$ enables calculation of K_d. In Eq. S36-S38, there are formulae that enable the determination of the slope and, therefore value of the dissociation constant for TnT complexes with bivalent, pentavalent and n-valent phage particles, respectively. It is important to mention that in the absence of TnT ($c_0^{TnT}=0$) both expressions on the right side equal zero, which may be treated as an additional data point next to experimental ones.

$$\frac{[TnT]}{K_d}=\frac{1}{K_d}\cdot c_0^{TnT}-\frac{c_0^{M13}\cdot\frac{\frac{[TnT]}{K_d}+2\cdot\left(\frac{[TnT]}{K_d}\right)^2}{1+\frac{[TnT]}{K_d}+\left(\frac{[TnT]}{K_d}\right)^2}}{K_d} \quad \text{(S36)}$$

$$\frac{[TnT]}{K_d}=\frac{1}{K_d}\cdot c_0^{TnT}-\frac{c_0^{M13}\cdot\frac{\sum_1^5\left(i\cdot\left(\frac{[TnT]}{K_d}\right)^i\right)}{1+\sum_1^5\left(\frac{[TnT]}{K_d}\right)^i}}{K_d} \quad \text{(S37)}$$

$$\frac{[TnT]}{K_d}=\frac{1}{K_d}\cdot c_0^{TnT}-\frac{c_0^{M13}\cdot\frac{\sum_1^n\left(i\cdot\left(\frac{[TnT]}{K_d}\right)^i\right)}{1+\sum_1^n\left(\frac{[TnT]}{K_d}\right)^i}}{K_d} \quad \text{(S38)}$$

In order to provide the best results, both c_0^{TnT} and c_0^{M13} should be similar to the expected K_d. A significant excess of TnT cause saturation of phage-displayed peptides, and the model provided above cannot be applied.
